# Pancreatic and Colonic Abscess Formation Secondary to HELLP Syndrome

**DOI:** 10.1155/2015/165435

**Published:** 2015-05-12

**Authors:** James M. O'Brien, Nicole Pursell, Fred Fumia

**Affiliations:** Department of Obstetrics and Gynecology, Jersey Shore University Medical Center, Neptune, NJ 07753, USA

## Abstract

Preeclampsia and the variant HELLP syndrome are systemic conditions associated with vascular changes resulting in vasoconstriction. Most commonly, patients present with elevated blood pressure and proteinuria, with a background of complaints such as headache, scotoma, and right upper quadrant pain. The systemic vascular changes experienced can target any organ system, oftentimes with more than one organ system being involved. We present the case of a patient admitted with HELLP syndrome who subsequently developed multisystem organ dysfunction, including placental abruption, disseminated intravascular coagulopathy, acute renal failure, colitis, abdominal ascites, pancreatitis, and the development of pancreatic and colonic abscesses.

## 1. Introduction

HELLP (hemolysis, elevated liver enzymes, and low platelets) syndrome is a condition on the spectrum of preeclampsia that is a serious complication occurring in 0.5% to 0.9% of all pregnancies and is seen in up to 20% of cases of severe preeclampsia [1]. The most common serious maternal complications are placental abruption, disseminated intravascular coagulation, and subsequent postpartum bleeding [1]. The systemic vascular insult that is experienced in preeclampsia and HELLP syndrome can target any organ system, often resulting in less commonly seen complications. In instances of systemic vascular insult, a patient on rare occasions may develop acute pancreatitis, acute renal failure, and acute colitis. Though infrequent, these serious complications attest to the mortality rate of 1.1% associated with HELLP syndrome [2]. There are few documented cases of patient's developing multisystem organ dysfunction secondary to the systemic vasoconstriction of HELLP syndrome, and there are no reported cases in the literature of a patient's postpartum course with HELLP syndrome being complicated by pancreatic and colonic abscess formation.

## 2. Case Report

A 20-year-old African American female, G1 at 33 weeks and 4 days of gestational age, was transferred to Jersey Shore University Medical Center with the complaint of frontal headaches associated with intermittent nausea and vomiting, which had been progressive in nature for two weeks. She was followed up by her obstetrician a few days prior in the office and was found to be normotensive, without proteinuria, and did not display any additional signs or symptoms of preeclampsia when seen and evaluated. The patient had no additional risk factors for the development of preeclampsia aside from her age and her ethnicity. Her nausea and vomiting were felt to be attributed to GERD, and she was prescribed a proton pump inhibitor.

The patient had initially presented to a local community hospital where she was found to have blood pressure ranging from 140–150/100–105 mmHg. Laboratory studies showed a creatinine of 2.3 mg/dL, AST 395 iU/L, ALT 400 iU/L, platelet count of 226,000/*μ*L, hematocrit of 35%, and no evidence of proteinuria on urinalysis. The diagnosis of partial HELLP syndrome was made based on elevated blood pressure, elevated liver enzymes, and the patient's symptoms. Fetal heart tracing was Category I with the baseline in the 120 s. The patient was maintained on magnesium sulfate for seizure prophylaxis and received a dose of betamethasone 12 mg IM for fetal lung maturity before being transferred to our medical center.

The patient's symptoms progressed to include right upper quadrant and epigastric pain. Blood pressure was noted to range from 140–145/80–90 mmHg on admission at our medical center. Shortly after admission, prior to an attempted induction of labor or obtaining laboratory data, an episode of fetal bradycardia to the 80s for greater than 10 minutes occurred despite resuscitative measures. The patient was urgently taken to the operating room where she was given general anesthesia and underwent a primary cesarean delivery. Delivery yielded a live born male infant, weighing 1640 grams, with APGARs of 7 and 9 and one and five minutes, respectively. The infant's birth weight placed it in the seventh percentile weight, based off the newborn preterm infant growth curve. Arterial cord blood pH was 7.027, with a base deficit of 15.2 mmol/L. Inspection of the placenta revealed approximately a 25% abruption. Pathology later confirmed this finding with evidence of multiple areas of infarct without evidence of inflammation.

Lab results obtained after delivery revealed an AST of 439 iU/L, ALT of 375 iU/L, creatinine of 2.43 mg/dL, LDH of 900 iU/L, uric acid of 9.4 mg/dL, and profound hyperglycemia with glucose of 459 mg/dL. Platelets were found to be 207,000/*μ*L. Additionally, a fibrinogen level was decreased at 134 g/L. A presumptive diagnosis of acute pancreatitis was made secondary to her hyperglycemia and was confirmed with serum amylase 284 U/L and serum lipase 210 U/L.

The patient's postoperative and postpartum course was complicated by multisystem organ dysfunction including coagulopathy which presented as acute onset of pressure dressing saturation and gingival bleeding several hours postoperatively. Her platelets fell to a nadir of 34,000/*μ*L with a hematocrit of 23.3% on postoperative day (POD) 5. She required IV labetalol and hydralazine intermittently for elevated blood pressure. She was started on a regular insulin sliding scale for her hyperglycemia. The patient continued to have abdominal pain and nausea/vomiting that was attributed to a postoperative ileus. She underwent a CT of the chest, abdomen, and pelvis on POD 6, which showed an enlarged pancreas consistent with pancreatitis, as well as diffuse colonic wall thickening suggestive of colitis, and diffuse pelvic and abdominal ascites. The patient required nasogastric tube placement and total parenteral nutritional support, due to her postoperative ileus, ascites, colitis, and pancreatitis.

The patient's postoperative course was additionally complicated by a superficial wound separation, which was positive for MRSA and required a wound vacuum; urinary retention requiring prolonged Foley catheter placement; and continued ascites in the setting of prolonged ileus. The patient began experiencing febrile episodes, with a maximum temperature of 102.5 degrees Fahrenheit on POD 10. Infectious disease was consulted, and the patient was placed on Vancomycin and Piperacillin/Tazobactam for empiric antibiotic coverage.

**Figure 1:**
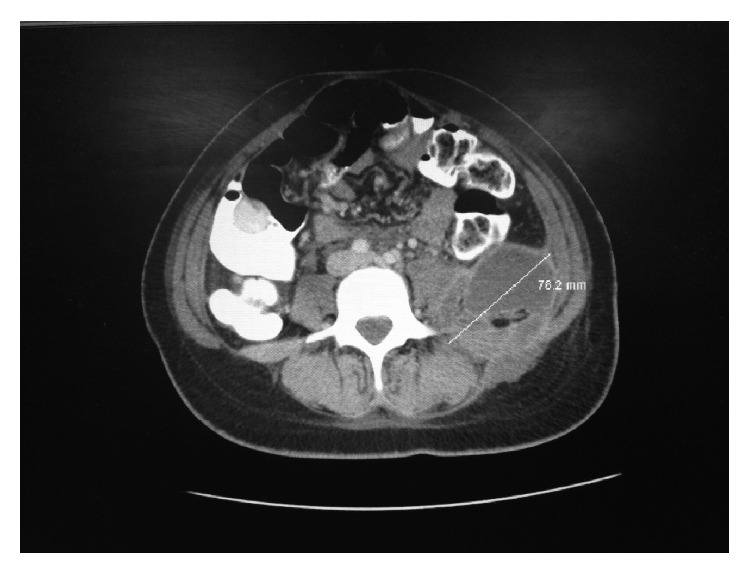
CT of abdomen/pelvic revealing a 7.6 × 5 cm left colonic abscess.

**Figure 2:**
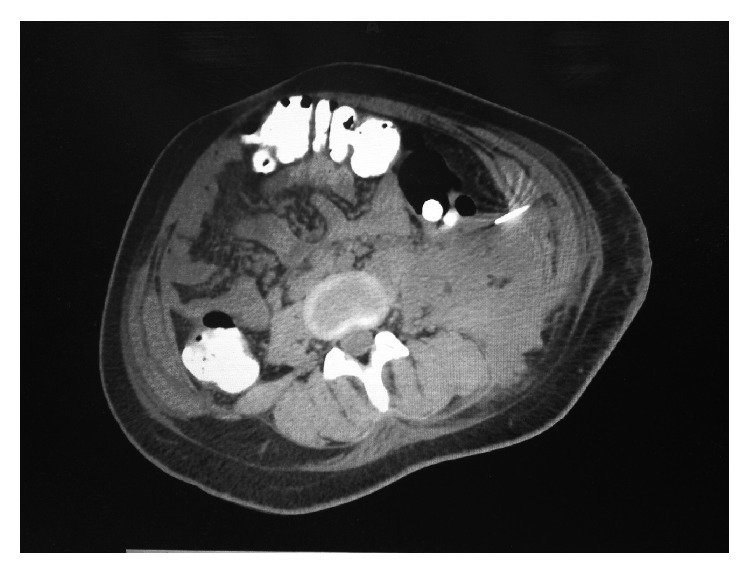
Interventional radiology placement of colonic abscess drain.

**Figure 3:**
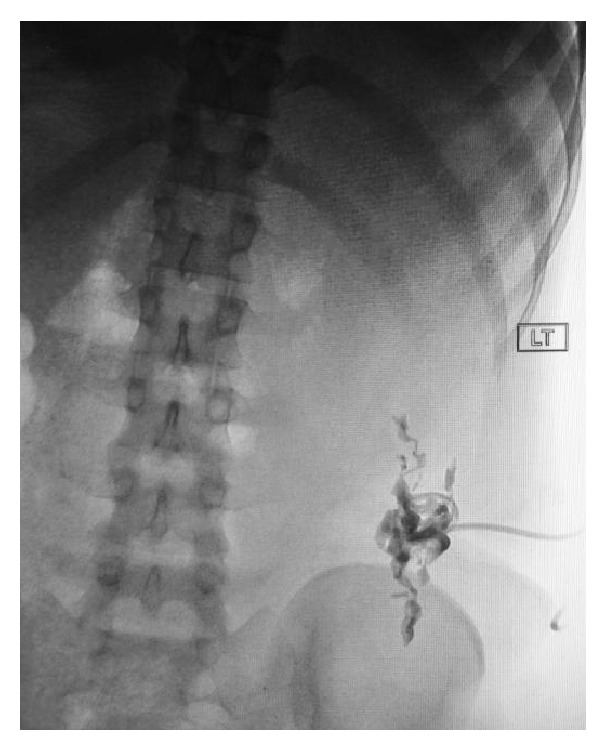
X-ray fluoroscopy confirming placement of colonic abscess drain.

Despite multiple courses with antibiotics and negative blood, urine, and repeat wound cultures, the patient continued to have intermittent febrile episodes. A repeat CT of the abdomen and pelvis on POD 15 revealed a 6 × 8 × 11 cm pancreatic abscess and a 7.6 × 5 cm left colonic abscess (Figure 1). The patient subsequently underwent CT guided drainage of the colonic abscess (Figures 2 and 3). Per recommendations by gastroenterology, there was no intervention made regarding the pancreatic abscess. After successful drainage of her colonic abscess, the patient ceased having febrile episodes. She was discharged home in stable condition on POD 21 after being afebrile for more than 24 hours. Her labs on discharger were as follows: WBC 10.4 K/*μ*L, hematocrit 24.5%, platelets 466,000/*μ*L, AST 46 iU/L, ALT 49 iU/L, LDH trending down at 405 iU/L, and glucose 86 mg/dL.

The patient was then readmitted to a neighboring community hospital on POD 34 with left flank pain. She was found to have recurrence of her left colonic abscess, which ran in the retroperitoneal space from the inferior aspect of the left kidney to the left groin, parallel to the iliopsoas and was 7 cm in diameter. The patient underwent an additional IR drainage of her colonic abscess, and cultures obtained grew multidrug resistant* Enterobacter cloacae*, sensitive to fluoroquinolones. She was discharged home on POD 39 with IR drain in place. She was maintained on moxifloxacin for 2 weeks and was followed up in 3 days for repeat CT evaluation and potential removal of the drain. She had successful removal of the drain and when seen and evaluated by her obstetrician several weeks later she was without complaint and continued to have normal vital signs as well as blood work.

## 3. Discussion

HELLP syndrome is a serious complication of pregnancy characterized by hemolysis, elevated liver enzymes, and a low platelet count occurring in up to 0.9% of pregnancies and carries a mortality rate of 1.1% [1, 2]. Serious maternal morbidity includes disseminated intravascular coagulopathy, placental abruption, acute renal failure, pulmonary edema, hepatic subcapsular hematoma, and retinal detachment [2]. Few cases of HELLP are complicated by multisystem organ failure.

Our patient presented with elevated liver enzymes and acute kidney injury. The presence of thrombotic microangiopathy involving the kidney can be so severe in HELLP syndrome that it can require hemodialysis [3]. The development of disseminated intravascular coagulopathy in our patient additionally complicated her immediate postoperative course, however this was no unexpected given the intraoperative findings of multiple placental infarcts. Activation of vascular endothelium and of platelets, hemolysis, and liver damage are the basic pathophysiological features of HELLP syndrome, each predisposing a patient to developing disseminated intravascular coagulopathy. In a retrospective cohort study, 38% of pregnant women with HELLP syndrome developed disseminated intravascular coagulopathy, most often following placental abruption [1]. Of note, our patient did not demonstrate thrombocytopenia on laboratory analysis until POD 4.

There are few documented cases of acute pancreatitis developing as the result of HELLP syndrome. Our patient developed profound hyperglycemia with elevated amylase and lipase levels on POD 1 and, as confirmed by CT imaging, the diagnosis of acute pancreatitis was made. Preeclampsia and HELLP syndrome are associated with endothelial injury, fibrin deposition in the vessel lumen, and increased platelet activation with platelet consumption. Platelet activation results in release of thromboxane A2 and serotonin, both vasoconstrictors. Platelet aggregation damages the endothelium and impairs the production of prostacyclin, a potent vasodilator [4]. The microvascular abnormalities and subsequent vasoconstriction associated with preeclampsia may cause not only HELLP syndrome, but also pancreatitis [5]. Recognizing that ischemia can damage not only the liver, but also the pancreas can result in improvement in the diagnosis and management of pancreatitis in preeclampsia [5]. Our patient developed not only acute pancreatitis, but also a pancreatic abscess secondary to the ischemic insult which occurred. The truly unique component to our patient's presentation was the development of colitis and a colonic abscess. There are no documented cases of abscess formation resulting from ischemic insult to the bowel secondary to preeclampsia or HELLP syndrome.

It is important to address the potential intrapartum, postpartum, and future medical complications in patients who have HELLP syndrome. The recurrence rate is 5–50% for preeclampsia and subsequent HELLP syndrome. The increased risk of preeclampsia with future pregnancies is as high as 19% in women without a history of preexisting hypertension and 75% in women with a history of hypertension [6]. Long term sequela associated with preeclampsia includes chronic hypertension, type II diabetes mellitus, stroke, and coronary artery disease [6].

We present a patient with HELLP syndrome complicated by placental abruption, acute renal failure, disseminated intravascular coagulopathy, acute pancreatitis with abscess formation, ascites, and acute colitis complicated by abscess formation which required CT guided drainage. Mortality rate associated with preeclampsia varied from 0 to 24%, with a delay in diagnosis of HELLP noted to significantly contribute to mortality [4]. While today there are predominately positive maternal outcomes in patients with HELLP syndrome, it is important to recognize that the vascular changes associated with the disease are capable of affecting multiple organ systems.
